# Qualitative and Quantitative Detection of CRISPR-Associated *Cas* Gene in Gene-Edited Foods

**DOI:** 10.3390/foods12193681

**Published:** 2023-10-07

**Authors:** Lin Ding, Xiaoli Xu, Xiaofu Wang, Xiaoyun Chen, Yuwen Lu, Junfeng Xu, Cheng Peng

**Affiliations:** 1 State Key Laboratory for Managing Biotic and Chemical Threats to the Quality and Safety of Agro-Products, Key Laboratory of Traceability for Agricultural Genetically Modified Organisms, Ministry of Agriculture and Rural Affairs, Zhejiang Academy of Agricultural Sciences, Hangzhou 310021, China; 2 State Key Laboratory for Managing Biotic and Chemical Threats to the Quality and Safety of Agro-Products, Key Laboratory of Biotechnology in Plant Protection of MARA and Zhejiang Province, Institute of Plant Virology, Ningbo University, Ningbo 315211, China

**Keywords:** gene editing technology, gene-edited foods, qualitative PCR, qPCR, *Cpf1*

## Abstract

Effective regulation of gene-edited products and resolution of public concerns are the prerequisites for the industrialization of gene-edited crops and their derived foods. CRISPR-associated protein, the core element of the CRISPR system, requires to be regulated. Thus, there is an urgent need to establish qualitative and quantitative detection methods for the *Cas* gene. In the present study, the primers and probes were designed and screened for *Cas12a* (*Cpf1*), which is the most commonly used target site in gene editing; we performed PCR system optimization, determined the optimal primer concentration and annealing temperature, and established qualitative PCR and quantitative PCR (qPCR) assays for detecting *Cpf1* in gene editing by specificity and sensitivity tests. In specificity testing, qualitative PCR and qPCR methods could 100% detect samples containing *Cpf1* DNA, while the detection rate of other samples without *Cpf1* was 0%. In the assay sensitivity test, the limit of detection of qualitative PCR was 0.1% (approximately 44 copies), and the limit of detection of the qPCR method was 14 copies. In the stability test, both the qualitative PCR and qPCR methods were repeated 60 times at their corresponding lowest detection limit concentrations, and the results were positive. Thus, the qualitative and quantitative assays for *Cpf1* are specific, sensitive, and stable. The method provides technical support for the effective monitoring of gene-edited products and their derived foods in the future.

## 1. Introduction

Gene editing technology can effectively edit target genes and is therefore a valuable tool in biological research [1,2,3]. The third-generation gene editing technology, namely clustered regularly interspaced short palindromic repeats (CRISPR), is a defense system in bacteria. The presence of these repeats was first discovered in *Escherichia coli* as early as 1987 by Ishino et al. [4], and, in 2000, Mojica et al. used computer analysis to find their prevalence in bacteria [5]. The CRISPR/Cas system has become one of the most popular gene editing techniques because of its simple vector construction process and high editing efficiency [6].

The Cas (CRISPR-associated) proteins of CRISPR/Cas systems bind to the transcription products of CRISPR to form complexes that function to cleave DNA sequences [7]. CRISPR/Cas systems are broadly classified into two major categories: type 1 and type 2. Type 1 includes type I, type III, and type IV, which require multiple Cas proteins. Type 2 includes types II, V, and VI, which require only a single scissor protein [8]. The common Cas9, Cas12a (Cpf1), and Cas13a belong to type 2; however, the Cas12 system substantially differs from the Cas9 system in the following aspects [9,10]: (1) Cas12a (Cpf1) requires only CRISPR RNAs (crRNA), while Cas9 requires trans-acting crRNA (tracr RNA) in addition to crRNA [11,12,13]; (2) Cas12a (Cpf1) has a higher number of target sites for editing because Cas12a (Cpf1) can recognize the protospacer adjacent motif (PAM) sites located at the 5′-end of the target that are rich in thymine (T) nucleotides, while Cas9 recognizes the PAM sites located at the 3′-end of the target that are rich in guanine (G) nucleotides; (3) Cas12a (Cpf1) can produce staggered ends, while Cas9 produces blunt ends [11,14]; (4) Cas9 has two nuclease domains, namely the RuvC nuclease domain and the HNH nuclease domain, while Cas12a (Cpf1) has only one RuvC-like nuclease domain; (5) in a large number of off-target analyses, Cas9 edits at unintended or random sites, which is not found in the majority of Cas12 systems [15]; and (6) because the CRISPR/Cpf1 system has a shorter crRNA and smaller Cas proteins, it can manage larger vector loads and is therefore more suitable for multitarget editing [9,11,16]. Cas12a specifically cuts target DNA, which is called cis cutting. In addition, Cas12a can form a ternary complex with crRNA and target DNA to cut the non-specific cutting activity of ssDNA, which is called trans cutting [17]. This feature is widely used in the field of nucleic acid detection [18,19,20].

The familiar Cas9 system has been widely used in gene-edited products. Following the introduction of Cas12a (Cpf1) with features lacking in Cas9, researchers are now using Cas12a (Cpf1) for gene editing in several species; however, there are no reports of products marketed with this system. Although some countries have allowed to market gene-edited plant-based foods, China is yet to establish a regulatory policy for gene-edited products. The academician Li Jiayang, a Chinese scientist, has proposed a regulatory framework for genome editing technologies; one of the most critical elements in this framework is whether the gene-edited foods contain exogenous DNA, such as nucleic acid endonucleases, in the pre-market stage [21], and, if exogenous nucleic acid sequences are introduced, they should be monitored by a similar regulatory strategy as that for transgenics. In 2021, we developed assays for *Cas9* [22]; however, much research remains to be conducted on qualitative and quantitative assays to detect *Cpf1*. Therefore, there is an urgent requirement to develop effective, specific, and sensitive screening and identification methods based on the *Cpf1* system. On the one hand, the research on the method to detect *Cpf1* is conducive to the screening and identification of *Cpf1* by researchers in the process of gene-edited crop breeding; on the other hand, the developed method can also be used as an important regulatory strategy for the future commercialization of gene-edited plant-based foods, which has a certain practical significance.

**Figure 1:**
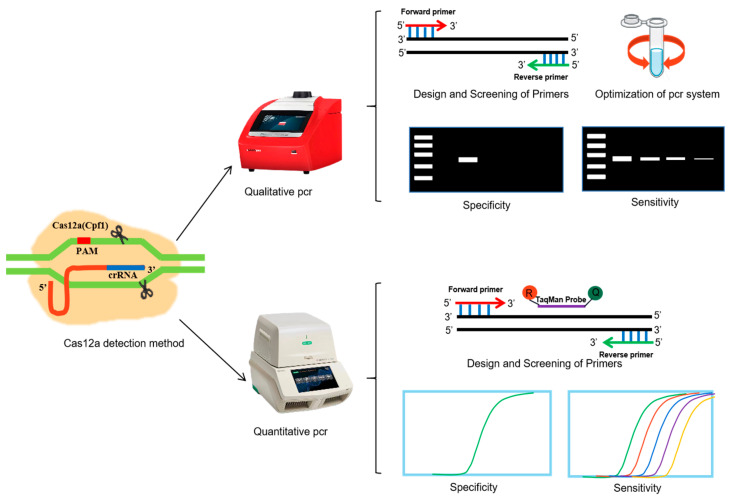
Schematic diagram of the experimental process.

In the present study, we establish a method to detect *Cpf1* based on the principle of gene editing, determine whether the test sample introduces exogenous DNA, such as *Cpf1*, and use the *Cpf1* introduced by gene-edited cotton as target sites. Qualitative PCR and quantitative PCR (qPCR) assays to detect *Cpf1* were established, and the system was optimized by designing and screening primers and analyzing the specificity and sensitivity of the method. The experimental flow is shown in Figure 1.

## 2. Materials and Methods

### 2.1. Materials

#### 2.1.1. Experimental

CRISPR-Cas12a (Cpf1) gene-edited cotton (*Gossypium hirsutum*) [23] and gene-edited rice (*Oryza sativa*) [24] were used as materials. Transgenic rice mixtures: TT51-1, KF-6, KF-2, KMD-1, M12, and KF-8; Transgenic soybean (*Glycine max*) mixtures: 356043, 305423, CV127, MON89788, A5547-127, and A2704-12; Transgenic maize (*Zea mays* L.) mixtures. Bt11, Bt176, MON810, MON863, GA21, NK603, T25, TC1507, MON89034, MON88017, 59122, MIR604, 3272, and MON87460; Transgenic oilseed rape (*Brassica napus*) mixtures: MS1, MS8, RF1, RF2, RF3, T45, Oxy235, and Topas19/2; Transgenic cotton mixture: MON1445, MON531, MON15985, LLCOTTON25, and MON88913. The above transgenic materials were purchased from the Science and Technology Development Center of the Ministry of Agriculture and Rural Development.

#### 2.1.2. Reagents

Plant genomic DNA kit (Tiangen Biochemical Technology Co., Ltd., Beijing, China); TaKaRa Taq, dNTP mixture, 10× PCR buffer (Mg^2+^ Plus), 6× Loading buffer (Bao Biological Engineering Co., Ltd., Dalian, China); DNA Ladder H1 (100~1000 bp), Agarose (low EEO), 10× TBE, Premixed Powder (Sangon Biotech Co., Ltd., Shanghai, China); 4SGelred (Sangon Biotech Co., Ltd., Shanghai, China); Fast Start Essential DNA Probes Master (Roche, Basel, Switzerland).

#### 2.1.3. Apparatus

KS12 biological safety cabinet (Thermo Fisher Scientific, Dreieich, Germany); Lab Dancer (IKA, Königswinter, Germany); Microfuge^®^ 16 centrifuge (BECKMAN, Brea, CA, USA); DK-S26 electric thermostatic water bath (Shanghai Senxin Experimental Instrument Co., Ltd., Shanghai, China); Nanodrop 2000 (ThermoFisher Scientific, Germany); Biometra Tadvanced 96 SG (Biometra, Göttingen, Germany); Lab cycler Gradient (SensoQuest, Göttingen, Germany); CFX96 Real-Time PCR system (Bio-RAD, Hercules, CA, USA); Automatic Gel Imaging analysis SystemZF-258 (Shanghai Jiapeng Technology Co., Ltd., Shanghai, China); Electrophoresis power supply EPS 301 (GE, Boston, MA, USA).

### 2.2. Methods

#### 2.2.1. Preparation of Samples

Gene-edited cotton with different mass fractions was prepared by using 100% gene-edited cotton and 100% non-gene-edited cotton in the following mass ratios: 10% (10 g of 100% gene-edited cotton, 90 g of 100% non-gene-edited cotton), 1% (10 g of 10% gene-edited cotton, 90 g of 100% non-gene-edited cotton), 0.1% (10 g of 1% gene-edited cotton, 90 g of 100% non-gene-edited cotton), 0.05% (10 g of 0.1% gene-edited cotton, 10 g of 100% non-gene-edited cotton) of DNA samples.

#### 2.2.2. DNA Extraction

Weighed 100 mg of various transgenic mixtures, non-gene edited cotton, and gene- edited cotton powder with mass fractions of 100%, 10%, 1%, 0.1%, and 0.05%, respectively, and extracted DNA using the plant DNA extraction kit according to the instructions.

#### 2.2.3. PCR Amplification

Qualitative PCR was performed using a 25 µL reaction system: 10× PCR buffer (Mg^2+^ Plus) 2.5 µL, dNTP mixture 2 µL, 0.5 µL each of forward and reverse primers (10 µmol/L) at the final concentration of 200 nmol/L, rTaq DNA polymerase (5 U/µL) 0.15 µL, DNA solution (50 ng/µL) 2 µL, the reaction mixture was supplemented with ddH_2_O to make up the volume to 25 µL. The reaction conditions were as follows: pre-denaturation at 95 °C for 5 min; denaturation at 95 °C for 30 s; annealing at 60 °C for 45 s; extension at 72 °C for 30 s; 35 cycles of amplification reaction, extension at 72 °C for 7 min; storage at 4 °C [25].

A 25 µL reaction system was used for qPCR: Fast Start Essential DNA Probes Master 12.5 µL, 1 µL each of 10 µmol/L forward and reverse primers at the final concentration of 400 nmol/L, probe (10 µmol/L) 0.5 µL at a final concentration of 200 nmol/L, DNA solution 2 µL, the reaction mixture was supplemented with ddH_2_O to make up the volume to 25 µL. The reaction conditions were as follows: pre-denaturation at 95 °C for 10 min; denaturation at 95 °C for 15 s; annealing and extension at 60 °C for 1 min; 40 cycles of amplification reaction; PCR amplification fluorescence signal collected at 60 °C.

##### Primer Design and Screening

Based on the sequence of Cpf1 in gene-edited cotton provided by the developer, three pairs of primers were designed using Primer Premier 5.0 based on the analysis of Cpf1 (Table S1), and the amplified fragment lengths analyzed by Primer Premier 5.0 were all in the range of 260–360 bp. The annealing temperatures were set as 52 °C, 53 °C, 54 °C, 56 °C, 57 °C, 59 °C, 61 °C, 63 °C, 65 °C, 66 °C, 67 °C, and 68 °C for the screening of the three primer pairs using the gene-edited cotton DNA solution.

The qPCR primers and probes were developed using Primer Express 3.0 software (Applied Biosystems, Waltham, MA, USA) (Table S2), and all primers were synthesized by Sangon Biotech (Shanghai) Co., Ltd. The DNA solution of gene-edited cotton was diluted in six gradients (190, 38, 7.6, 1.52, 0.304, and 0.0608 ng/µL), and the standard curves were made for the designed primers to verify the efficiency of the primers.

##### Qualitative PCR System Optimization

The qualitative PCR system was optimized by setting the primer concentrations to 0.1, 0.2, 0.3, 0.4, and 0.5 μmol/L. The amplification products were detected by 2% agarose gel electrophoresis, and the best primer concentration was the one with brighter amplification bands.

##### Specificity Analysis

PCR amplification was performed using 1% of gene-edited cotton, 6 other transgenic rice mixes, 6 common transgenic soybean mixes, 14 common transgenic maize mixes, 8 common transgenic oilseed rape mixes, 5 common transgenic cotton mixes, and non-gene-edited cotton DNA as templates, and qualitative PCR amplification products were electrophoresed on a 2% agarose gel at 160 V for 30 min. The results were observed by gel imaging system. qPCR amplification results were observed by amplification curve.

##### Sensitivity Analysis

Qualitative PCR sensitivity assay: DNA of gene-edited cotton with mass fractions of 100%, 10%, 1%, 0.1%, and 0.05% were used as templates and amplified with primers, and their amplification products were electrophoresed on 2% agarose gel at 160 V for 30 min, and the results were observed using a gel imaging system.

qPCR sensitivity assay: 100% genomic DNA of gene-edited cotton was diluted to 190, 38, 7.6, 1.52, 0.304, 0.0608 ng/µL for qPCR amplification, and the results of the amplification curve were observed.

## 3. Results

### 3.1. Designing and Screening of Primers

**Figure 2:**
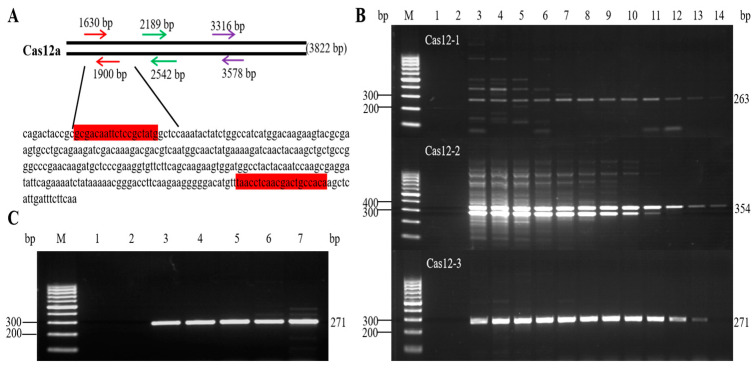
(**A**) Sequence of *Cpf1*. Highlight: Forward primer and reverse primer. (**B**) Screening of the primer pairs. For primers Cas12-1, Cas12-2, and Cas12-3, the amplified bands were 263, 354, and 271 bp, respectively. 1: Blank control; 2: Negative control; 3–14: Annealing temperatures 52 °C, 53 °C, 54 °C, 56 °C, 57 °C, 59 °C, 61 °C, 63 °C, 65 °C, 66 °C, 67 °C, and 68 °C; M: DNA molecular weight marker Ladder H1 (100–1000 bp); the same is used below. (**C**) Screening of PCR conditions for primer Cas12-3. 1: Blank control; 2: Negative control; 3–7: Primer concentrations of 0.1, 0.2, 0.3, 0.4, and 0.5 μmol/L.

Based on the sequence of *Cpf1* (GenBank Accession No.: OK557998.1) shown in Figure 2A, three pairs of primers were developed using Primer Premier 5.0 to assess the specificity, sensitivity, and efficiency of the method.

In the experiment, the gene-edited cotton DNA containing *Cpf1* was used as the template, and an annealing temperature gradient experiment was performed to determine the optimum annealing temperature. The annealing temperatures were set at different temperatures. The results of PCR amplification showed that some of the products amplified by primer Cas12-1 had nonspecific amplification products, and a single band was present at the annealing temperature of 59–68 °C; however, the bands were weak. The amplified product of primer Cas12-2 had more nonspecific amplification products, and, although there was a single band at 66–68 °C, this band was weak. The amplification product of primer Cas12-3 had a single and clear band and no primer dimer formation, and the highest amplification efficiency was achieved at the annealing temperature of 59–63 °C (Figure 2B). On the basis of the experimental results, the final identified primer was Cas12-3, and the amplified band size was 271 bp. The optimal annealing temperature range was 59–63 °C.

### 3.2. Optimization of the Qualitative PCR Reaction System and Amplification Conditions

System optimization experiments were performed with different primer concentrations. The results (Figure 2C) showed that the amplified bands became significantly stronger with increasing primer concentrations at the annealing temperature of 60 °C; however, nonspecific amplification products appeared at higher primer concentrations. The sensitivity and specificity of the assay were evaluated, and the primer concentration of 0.3 μmol/L was found to yield a high level of amplification efficiency without nonspecific amplification products and primer dimer formation. Thus, the optimum annealing temperature of 59–63 °C was determined in the primer design and screening experiments mentioned in Section 2.1. The PCR reaction mixture contained PCR buffer (Mg^2+^ Plus) 2.5 µL, dNTP mixture 2 µL, Cas12-F3 and Cas12-R3 (10 µmol/L) 0.75 µL each at the final concentration of 300 nmol/L, r*Taq* DNA polymerase (5 U/µL) 0.15 µL, and DNA solution (50 ng/µL) 2 µL; the reaction mixture was supplemented with ddH_2_O to make up the volume to 25 µL. The reaction conditions were as follows: pre-denaturation at 95 °C for 5 min; denaturation at 95 °C for 30 s; annealing at 59–63 °C for 45 s; extension at 72 °C for 30 s; 35 cycles of amplification reaction, extension at 72 °C for 7 min; and storage at 4 °C.

### 3.3. Specificity Analysis of the Qualitative PCR Method

**Figure 3:**
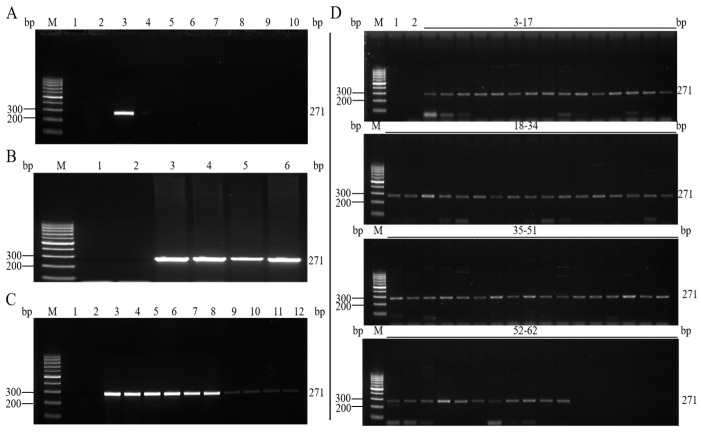
(**A**) Specificity assay of the qualitative PCR method for gene-edited cotton. 1: Blank control; 2: Negative control; 3: Gene-edited cotton with mass fraction of 1%; 4: Transgenic cotton mixture; 5: Transgenic soybean mixture; 6: Transgenic rice mixture; 7: Transgenic corn mixture; 8: Transgenic rapeseed mixture; 9: Non-gene-edited cotton; 10: Gene-edited rice. (**B**) Detection results of gene-edited cotton. 1: Blank control; 2: Negative control; 3: Gene-edited cotton-1; 4: Gene-edited cotton-2; 5: Gene-edited cotton-3; 6: Gene-edited cotton-4. (**C**) Sensitivity assay of the qualitative PCR method for gene-edited cotton. 1: Blank control; 2: Negative control; 3–4: Gene-edited cotton with mass fraction of 10%; 5–6: Gene-edited cotton with mass fraction of 5%; 7–8: Gene-edited cotton with mass fraction of 1%; 9–10: Gene-edited cotton with mass fraction of 0.1%; 11–12: Gene-edited cotton with mass fraction of 0.05%. (**D**) Stability test of PCR detection method for gene-edited cotton. 1: Blank control; 2: Negative control; 3~62: The results of 60 independent amplifications of gene-edited cotton DNA with 0.1% mass fraction.

To determine the specificity of the established qualitative PCR assay, the DNA of mixed samples of other transgenic samples, the DNA of rice samples edited by the Cas9 system, and the DNA of non-gene-edited cotton were used as templates for PCR amplification [26], and the experimental results are shown in Figure 3A. Only 1% of the gene-edited cotton samples amplified to the expected DNA fragments, while, in the other samples, no bands of the expected size were amplified. This finding indicates that the established method to detect *Cpf1* is highly specific. Next, four different gene-edited cotton samples were amplified using primer Cas12-3, and the experimental results are shown in Figure 3B. Except for the blank control and the negative control, all the gene-edited cotton line samples amplified the target fragment of the expected size.

### 3.4. Sensitivity Analysis of the Qualitative PCR Method

In the sensitivity analysis of the qualitative PCR method, DNA from gene-edited cotton samples with mass fractions of 10%, 5%, 1%, 0.1%, and 0.05% was used as a template for qualitative PCR amplification. The results (Figure 3C) showed that the PCR products showed weaker bands as the content of gene-edited cotton DNA decreased; however, bands still appeared in the PCR amplification at levels as low as 0.05%, thus indicating that the sensitivity of the method could reach 0.1%. To further determine the lower limit of stable detection of the method at 0.1%, 60 qualitative PCR amplifications were performed using gene-edited cotton genomic DNA with a mass fraction of 0.1% as the template. The results are shown in Figure 3D. All the 60 repeated qualitative PCR experiments yielded targeted amplification products, thus meeting the requirements for determining the qualitative detection limit of gene editing components [27]. Therefore, the detection limit of the method was taken as 0.1%. The concentration of the DNA template in the PCR reaction system was 50 ng/µL, and the size of the cotton haploid genome was 2118 Mbp; thus, the limit of detection (LOD) of this method was approximately 44 copies.

### 3.5. Primer Design and Screening for qPCR

**Figure 4:**
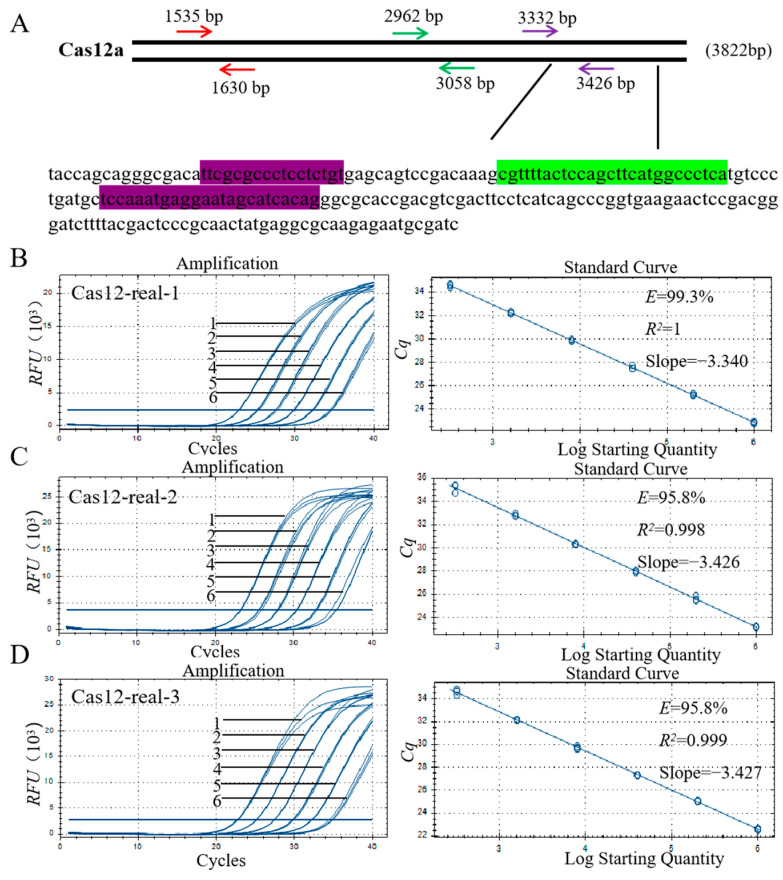
(**A**) Sequence of *Cpf1*. Highlight: Forward primer, reverse primer, and probe. (**B**) Amplification curve and standard curve of Cas12-real-1. (**C**) Amplification curve and standard curve of primer Cas12-real-2. (**D**) Amplification curve and standard curve of primer Cas12-real-3. 1–6: DNA concentrations were 190, 38, 7.6, 1.52, 0.304, and 0.0608 ng/µL; RFU: Relative fluorescence units; *Cq*: quantification cycle; the same is shown below.

The present study further developed the quantitative detection method for *Cas12a (Cpf1)*. On the basis of the *Cpf1* sequence (Figure 4A), three pairs of primer and probe combinations were designed using Primer Express 3.0 software (Applied Biosystems, USA) (Table S2).

Three pairs of primers and probes were used to amplify gene-edited cotton genomic DNA with a mass fraction of 100% and the amplification templates were diluted to different concentrations. Standard curves were constructed for the three pairs of primers and probes to verify the primer amplification efficiency, and the results are shown in Figure 4B–D. The amplification efficiency was calculated according to the slope of the standard curve: E = 10^−1/slope^−1. The quantitative detection of gene editing exogenous components requires the slope of the standard curve to be in the range of −3.6 ≤ slope ≤ −3.1, which implies that the amplification efficiency is 90–110%, and the correlation coefficient (R^2^) is ≥0.98 [28]. The results showed that the slope of Cas12-real-1 was −3.340, the amplification efficiency E was 99.3%, and the correlation coefficient R^2^ was 1. The slope of Cas12-real-2 was −3.426, the amplification efficiency E was 95.8%, and the correlation coefficient R^2^ was 0.998. The slope of Cas12-real-3 was −3.427, the amplification efficiency E was 95.8%, and the correlation coefficient R^2^ was 0.999. Thus, all indicators of the three primer pairs met the requirements of the standard curve for the quantitative detection of exogenous components of gene editing. However, according to the extent of amplification, the primers and probes of Cas12-real-1 were superior to those of Cas12-real-2 and Cas12-real-3, and the primer for the qPCR assay was finally determined to be Cas12-real-1.

### 3.6. Specificity Assay for the qPCR Method

**Figure 5:**
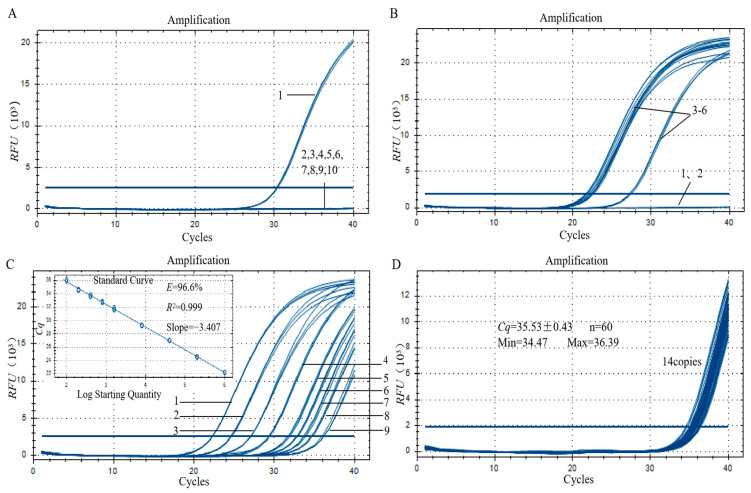
(**A**) Specificity assay of the qPCR detection method for gene-edited cotton. 1: Gene-edited cotton with mass fraction of 1%; 2: Blank control; 3: Negative control; 4: Transgenic cotton mixture; 5: Transgenic soybean mixture; 6: Transgenic rice mixture; 7: Transgenic corn mixture; 8: Transgenic rapeseed mixture; 9: Non-gene-edited cotton; 10: Gene-edited rice. (**B**) Amplification curves of gene-edited cotton. 1: Blank control; 2: Negative control; 3: Gene-edited cotton-1; 4: Gene-edited cotton-2; 5: Gene-edited cotton-3; 6: Gene-edited cotton-4. (**C**) Linear interval test for the qPCR Method; 1–9: DNA concentrations were 160, 32, 6.4, 1.28, 0.256, 0.128, 0.064, 0.032, and 0.016 ng/µL. (**D**) LOD test for the qPCR method.

To determine the specificity of the established qPCR assay for gene-edited cotton, the DNA of mixed samples of other transgenic crops, the DNA of rice samples edited by the Cas9 system, and the DNA of non-gene-edited cotton were used as templates for qPCR amplification. By using 1% of gene-edited cotton as a positive control and both negative and blank controls, no specific amplification curve was obtained in any of the samples, except for the positive control. As shown in Figure 5A, the amplification results indicate that Cas12-real-1 has a high specificity to detect *Cpf1*. Next, four samples of a different gene-edited cotton were selected and amplified with Cas12-real-1. Amplification curves were obtained in all positive samples (Figure 5B).

### 3.7. Sensitivity Analysis of the qPCR Method

In the sensitivity assay of the qPCR method, 100% genomic DNA of gene-edited cotton was diluted to different concentrations for qPCR. The standard curve was then plotted. As shown in Figure 5C, the slope of the standard curve was −3.407, the amplification efficiency E was 96.6%, and the correlation coefficient R^2^ was 0.999. All the correlation values were within the range (−3.6 ≤ slope ≤ −3.1, amplification efficiency E was 90–110%, and R^2^ ≥ 0.98). This finding indicates that the method has good linearity in this template concentration range. The qPCR method detected 0.016 ng/µL of the genomic DNA of gene-edited cotton, which corresponds to 14 copies of the gene-edited cotton genome. We repeated the experiment 60 times with 0.016 ng/µL DNA, and typical amplification curves were obtained (Figure 5D). The results showed that the *Cq* value of 60 replicates was 35.53 ± 0.43, the SD value was 0.43, and the relative standard deviation (RSD) value was 1.2%, which determined the detection limit of the qPCR method as 14 copies of the gene-edited cotton genome.

## 4. Discussion

Presently, with a rapidly growing population and a surge in demand for food, modern agriculture needs to make sustainable developments for producing high quality and quantity of crops that can withstand global climatic change and other biological factors. In the past few years, CRISPR technology has greatly accelerated the pace of crop research and breeding [6]. The Cas9 system has been widely used in various fields. The advantages of the Cas12 system can overcome the limitations of the Cas9 system, and, thus, it is expected that the Cas12 system could become a substitute for the Cas9 system. CRISPR/Cpf1 technology enables breeders to improve crop yield and quality in an efficient and accurate manner [29,30]. Currently, researchers have used the Cas12 system to edit various plants, such as *Arabidopsis thaliana* [31], cotton [23], rice [30], maize [6], *Glycine max* var. [29], tomato [32], *Chlamydomonas reinhardtii* [33], and citrus [34]. The CRISPR/Cas12 system also has strong advantages in editing bacteria, and many bacterial species are widely used in life science industries, such as pharmaceutical [35], biotechnology, and cosmetics [36]. Many byproducts of microbial metabolism, such as enzymes and antibiotics, are used by humans [37]; hence, studying and modifying the genome to meet research needs are required to fully utilize the true potential of microorganisms. In gene-edited bacteria, the Cas12a (Cpf1) system has many advantages that enable nucleotide substitutions, deletions, and insertions in the genome with greater efficiency [38,39]. In the research on the application of the Cas12a (Cpf1) system for editing mammalian cell lines, Cas12a (Cpf1) can reverse the disease status of cell lines [40,41,42], and this system is expected to be a safe and effective gene editing tool that can be used clinically.

The nucleic acid detection function of Cpf1 is also highly valued by researchers, and Hour Low-cost Multipurpose highly Efficient System (HOLMES) can use Cpf1 to efficiently and rapidly detect target DNA if it is present in the sample. Cpf1/crRNA of HOLMES forms a ternary complex with the target DNA and triggers the trans-cleavage of the nontargeted ssDNA; thus, the breakage of the reporter DNA causes the fluorescent group to generate fluorescent signals. This detection tool opens a new window to diagnose human diseases. Previous studies have shown that HOLMES can be used to detect DNA and RNA viruses, such as pseudorabies virus and Japanese encephalitis virus, and for food and environmental monitoring [43]. In the era of rapid development of gene editing, the Cas12a (Cpf1) system is expected to become a more popular scissor protein; however, the effective monitoring and regulation of gene editing technology remains a critical issue. Thus far, no research has been reported for the detection method of *Cpf1*.

In the present study, the presence or absence of exogenous DNA such as *Cpf1* in the sample was detected to identify whether the sample was a gene-edited product and the derived foods based on the principle of gene editing. In this study, *Cpf1* introduced by gene-edited cotton was used as the target site to design primer pairs for specific qualitative PCR and qPCR amplification to ensure the detection of exogenous sequences. Two detection methods were established: qualitative PCR and qPCR. The advantages of qualitative PCR are low instrumentation requirements, simple operation, and low cost; however, the method is time-consuming. The qPCR method has the advantages of real-time analysis, high sensitivity, less time consumption, and detection of low levels of gene-edited components. Thus, the combination of the two methods in a complementary assay could yield accurate and rapid detection [28], It will also provide some technical support to establish a detection and identification system of gene-edited foods in the future.

The method of *Cpf1* detects different exogenous genes compared to the previously studied *Cas9* assay. In experiments such as primer design and screening, sensitivity, and detection limit, the detection of *Cpf1* is more sensitive and has a lower detection limit compared to *Cas9*. In terms of primer selection, the analysis of *Cpf1* provides more primer sequences, and only the optimal primers and probes were selected in the manuscript. In fact, these primers and probes can complete the detection of *Cpf1*. Secondly, in qualitative PCR experiments, the sensitivity of the *Cpf1* assay (44 copies) was higher than that of the *Cas9* assay (65 copies). In the qPCR method, the amplification efficiency E of the optimal primer for *Cpf1* was 99.3%, and the correlation coefficient R^2^ was 1, and the amplification efficiency E of the optimal primer for *Cas9* was 95.2%, R^2^ = 0.999. The detection limit for *Cpf1* was 14 copies, while the detection limit for *Cas9* was 16 copies [22]. In summary, the detection method of *Cpf1* is a highly sensitive and applicable method.

## 5. Conclusions

In the present study, qualitative PCR and qPCR assays were established to detect the specificity of *Cpf1*, which was used in gene-edited cotton as the target site. The detection limit of the qualitative PCR assay was 0.1% (approximately 44 copies), and the LOD of the qPCR method was 14 copies. The two methods established in this study have high specificity, good sensitivity, and reproducibility; can yield stable and reliable results; and are suitable for qualitative and quantitative analysis of the exogenous components of *Cpf1* in some gene-edited foods and primary screening for *Cpf1* detection.

## Data Availability

Data are contained within the article.

## Supplementary Materials

The following supporting information can be downloaded at: https://www.mdpi.com/article/10.3390/foods12193681/s1, Table S1: The information of primers used in qualitative PCR; Table S2: The information of primers and probes used in qPCR.
