# Supplementary material for: Qualitative and Quantitative Detection of CRISPR-Associated Cas Gene in Gene-Edited Foods
Source: Foods. 2023 Oct 7;12(19):3681. doi: 10.3390/foods12193681 (PMC10572612; doi:10.3390/foods12193681)
Supplement: Supplementary file 1 [file foods-12-03681-s001.zip › foods-2597461-supplementary.pdf]

Supplementary Table S1. The information of primers used in qualitative PCR.

Primer	Sequence(5′-3′)	Amplication length/bp
Cas12-F1	TACCAGCAGGGCGACATTC	263
Cas12-R1	CAGAGCACTTTCCTGGCGATA	
Cas12-F2	AGAACAACCACGGGCAAATC	354
Cas12-R2	CCTTCCCATCCACGACCAC	
Cas12-F3	GCGACAATTCTCCGCTATG	271
Cas12-R3	TGTGGCAGTCGTTGAGGTTA	

Supplementary Table S2.The information of primers and probes used in Qpcr.

Primer	Sequence(5′-3′)	Amplication length/bp
Cas12-real-F1	TTCGCGCCCTCCTCTGT	95
Cas12-real-R1	TGTGATGCTATTCCTCATTTGGA	
Cas12-real-P1	FAM-CGTTTTACTCCAGCTTCATGGCCCTCA-BHQ1	
Cas12-real-F2	ACCCAGAACGGGTTTATTTTCTACA	97
Cas12-real-R2	AGGTATACTTTGTCTTCAGGAGGTTCA	
Cas12-real-P2	FAM-CTCACCTCCAAGATTGACCCGAGCAC-BHQ1	
Cas12-real-F3	CACAGAAGCCGTACTCCAAA	96
Cas12-real-R3	CGCGGTAGTCTGTCTCTTTATC	
Cas12-real-P3	FAM-AAGCTGTACTTCCAGAACCCGCAA-BHQ1	
